# Environmental filtering and biotic interactions act on different facets of the diversity of benthic assemblages associated with eelgrass

**DOI:** 10.1002/ece3.10159

**Published:** 2023-11-27

**Authors:** Alexandre Muller, Stanislas F. Dubois, Aurélien Boyé, Ronan Becheler, Gabin Droual, Mathieu Chevalier, Marine Pasquier, Loïg Roudaut, Jérôme Fournier‐Sowinski, Isabelle Auby, Flávia L. D. Nunes

**Affiliations:** ^1^ IFREMER Centre de Bretagne, DYNECO Laboratoire d'Ecologie Benthique Côtière Plouzané France; ^2^ DECOD (Ecosystem Dynamics and Sustainability), IFREMER, INRAE Institut Agrocampus Ouest Nantes France; ^3^ CNRS, Centre d'Écologie et des Sciences de la Conservation (CESCO) Station de Biologie Marine MNHN Concarneau France; ^4^ IFREMER, Laboratoire Environnement Ressources d'Arcachon Arcachon France

**Keywords:** benthic communities, Beta diversity, biodiversity, foundation species, functional diversity, structural equation modeling, *Zostera marina*

## Abstract

Eelgrass supports diverse benthic communities that ensure a variety of ecosystem functions. To better understand the ecological processes that shape community composition in eelgrass at local and regional scales, taxonomic and functional α‐ and β‐diversity were quantified for communities inhabiting five meadows in France. The extent to which environmental factors affected local and regional benthic communities was quantified by considering their direct and indirect effects (through morphological traits of eelgrass) using piecewise structural equation modeling (pSEM). Communities supported by eelgrass had higher species abundances, as well as taxonomic and functional diversity compared to nearby bare sediments. No significant differences were found between communities from the center relative to the edges of meadows, indicating that both habitats provide similar benefits to biodiversity. The presence of a few abundant species and traits suggests moderate levels of habitat filtering and close associations of certain species with eelgrass. Nevertheless, high turnover of a large number of rare species and traits was observed among meadows, resulting in meadows being characterized by their own distinct communities. High turnover indicates that much of the community is not specific to eelgrass, but rather reflects local species pools. pSEM showed that spatial variation in community composition (β‐diversity) was primarily affected by environmental conditions, with temperature, current velocity, and tidal amplitude being the most significant explanatory variables. Local richness and abundance (α‐diversity) were affected by both environment and morphological traits. Importantly, morphological traits of *Zostera marina* were also influenced by environmental conditions, revealing cascading effects of the environment on assemblages. In sum, the environment exerted large effects on community structure at both regional and local scales, while plant traits were only pertinent in explaining local diversity. This complex interplay of processes acting at multiple scales with indirect effects should be accounted for in conservation efforts that target the protection of biodiversity.

## Graphical abstract

Combining taxonomic and trait‐based approaches with structural equation modeling, this study explored the mechanisms governing community assembly in five meadows located over a distance of 800 km along the French coast in the Northeast Atlantic. Meadows appear to harbor subsets of species from the regional species pool, rather than harboring eelgrass‐specific assemblages. Given that eelgrass enhances the diversity and abundance of species found in neighboring habitats, conservation programs should consider ecosystem‐level protection spanning multiple habitats, including eelgrass, to maximize the protection of biodiversity.

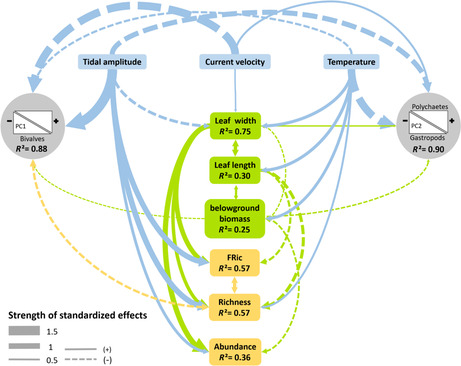

## 1 INTRODUCTION

Marine biodiversity contributes to healthy and resilient ecosystems but is currently threatened by a multitude of human activities such as climate change, overharvesting, and pollution (Isbell et al., 2017). To make informed decisions about seascape management and conservation (Kavanaugh, 2019), it is essential to understand the processes that control the distribution of diversity across marine habitats at both local and regional scales (Thompson et al., 2020). At broad geographic scales, evolutionary, geological, and colonization histories define a regional species pool, referred to as γ‐diversity (Mittelbach & Schemske, 2015; Whittaker, 1972). At finer scales, biotic interactions (predation, competition) and abiotic conditions within habitats filter species from the regional pool leading to what is known as α‐diversity (Thompson et al., 2020; Whittaker, 1972). Interactions between local and regional processes generate spatial and temporal gradients in community structure known as β‐diversity (Anderson et al., 2011; Whittaker, 1972). Examining the different components of diversity is essential for determining the factors that structure communities of a given habitat type. For instance, β‐diversity can be partitioned into species turnover (the replacement of species or functional strategies in one assemblage compared with another) and nestedness (differences in richness when one assemblage is a subset of another; Baselga, 2010, 2012; Legendre, 2014). Considering these two components of β‐diversity helps to identify the processes that lead to differences in assemblages, such as niche differentiation (high turnover) or niche filtering (high nestedness; Loiseau et al., 2017; Villéger et al., 2013).

Beyond taxonomy, it is now widely recognized that the integration of functional information based on species traits provides a complementary understanding of the processes structuring communities along environmental gradients (Mori et al., 2018; Pavoine & Bonsall, 2011). Comparing taxonomic and functional diversity can provide insights into the ecological processes that shape community composition (Mori et al., 2018; Villéger et al., 2010) and the impact of biodiversity loss on ecosystem functioning (Burley et al., 2016; Cadotte et al., 2011). For instance, environmental filtering is expected to lead to lower functional diversity than expected from taxonomic diversity (functional underdispersion), while competition for resources will often promote trait differentiation, leading to the opposite pattern (functional overdispersion; Münkemüller et al., 2020; Perronne et al., 2017).

Biodiversity is typically greater in structurally complex compared with homogeneous habitats (Lapointe & Bourget, 1999). Foundation species (Dayton, 1972) not only complexify the habitat but also control the availability of resources for other organisms (Ellison, 2019; Sarà, 1986). By modifying habitat, foundation species can influence community assembly and its long‐term persistence through numerous mechanisms such as niche partitioning (Willis et al., 2005), altering competitive and predator–prey interactions (Costello et al., 2015), or providing refuge from physical stressors (Bulleri et al., 2016; Jurgens & Gaylord, 2018). Because community composition can vary greatly within habitats across environmental gradients (Boström et al., 2006; Boyé et al., 2017), studying the effect of habitat complexity on the associated communities improves our understanding of the processes structuring biodiversity at various geographic scales (Airoldi et al., 2008). While foundation species exert many direct effects on the communities they support, less is known about the indirect effects that they may exert on communities (e.g., by modifying resource availability), with potentially important indirect effects being left unaccounted (Miller et al., 2018).

*Zostera marina* (Linnaeus, 1753), commonly called eelgrass, is a flowering marine plant that occurs from temperate to subarctic regions (Green & Short, 2004), forming meadows that are recognized as being among the most important coastal marine ecosystems on the planet (Unsworth et al., 2022). Eelgrass is a foundation species, providing essential functions and services including coastal protection, erosion control, nutrient cycling, water purification, carbon sequestration, as well as food and habitat for a variety of species (Barbier et al., 2011; Cullen‐Unsworth & Unsworth, 2013; Fourqurean et al., 2012). Eelgrass can have a strong influence on the spatial distribution of associated fauna by altering the hydrodynamics of the marine environment (Fonseca & Fisher, 1986), providing abundant resources, available surface area, and increased ecological niches (Duffy, 2006). Eelgrass meadows are dynamic habitats that are constantly changing in space and time (Clarke & Kirkman, 1989). Wave action may remove sediments in the more exposed parts of a meadow, leading to the uprooting of shallow rhizomes (Fletcher & Fletcher, 1995; Orth et al., 2006), while an increase in sediment input may bury the meadow (Terrados et al., 1997). Different parts of the meadows may experience different levels of environmental disturbances, with the central core being less likely to be exposed to currents and uprooting, while these processes may occur frequently at the edges of the meadows. Edges may have impoverished communities as a result of instability or they may be ecotones (transition zones between meadows and bare sediment) that harbor species from both habitats, thus having higher diversity (Arponen & Boström, 2012; Fahrig, 2020; Fahrig et al., 2019; Kark & van Rensburg, 2006). While the presence of eelgrass is typically associated with high taxonomic diversity (Boström & Bonsdorff, 1997; Hily & Bouteille, 1999), diversity gradients within meadows can vary widely among regions or taxonomic groups (Boström et al., 2006; Wong & Dowd, 2015 and references therein), warranting further investigation.

The variability of eelgrass structure in relation to its physical environment is fairly well understood (Boyé et al., 2022; Frederiksen et al., 2004), as is the effect of the environment on the community structure (Blake & Duffy, 2012; Yeager et al., 2019). However, understanding how the traits of the foundation species are affected by their environment and how these two in turn affect community structure has proven more difficult. Furthermore, the relative contributions of biotic and abiotic factors in explaining biodiversity at multiple spatial scales have been poorly examined or quantified (Bowden et al., 2001; Hovel et al., 2002; Turner et al., 1999). Indeed most of the potential cascading effects of the environment on foundation species, and subsequently on associated fauna studied to date involve the loss or replacement of foundation species (Airoldi et al., 2008; Ellison et al., 2005; Pessarrodona et al., 2019; Sorte et al., 2017). Understanding how the environment affects biodiversity directly or indirectly by modifying traits of the foundation species may help to better understand the biotic and abiotic factors that shape communities associated with eelgrass.

In this study, the taxonomic and functional diversity of assemblages associated with five *Z. marina* meadows occurring over a distance of 800 km along the coast of France were investigated with the objective of determining which factors control community composition within this habitat. To this end, we examined α‐ and β‐diversity of assemblages based on species‐ and trait‐based descriptors, with a focus on polychaetes, gastropods, and bivalves; three diverse groups exhibiting a wide range of ecological strategies (Jumars et al., 2015) and having central roles in ecosystem functioning including bioturbation or cycling of organic matter (Duffy et al., 2015; Queirós et al., 2013). Specifically, we asked the following questions: (i) Are there differences in terms of abundance, as well as in species and trait diversity within eelgrass meadows (i.e., core vs. edge) and with respect to the adjacent bare sediment? (ii) Are there differences in the taxonomic and functional diversity of assemblages from different meadows? (iii) What are the processes explaining the α‐diversity and β‐diversity of eelgrass‐associated fauna? (iv) Finally, what are the direct and indirect effects of environmental factors on the structure of assemblages associated with eelgrass? By addressing these questions, we aim to improve our understanding of community assembly rules at work in *Z. marina* meadows, which will ultimately help guide conservation measures in this important habitat.

## 2 METHODS

### 2.1 Study area and sampling methods

**FIGURE 1:**
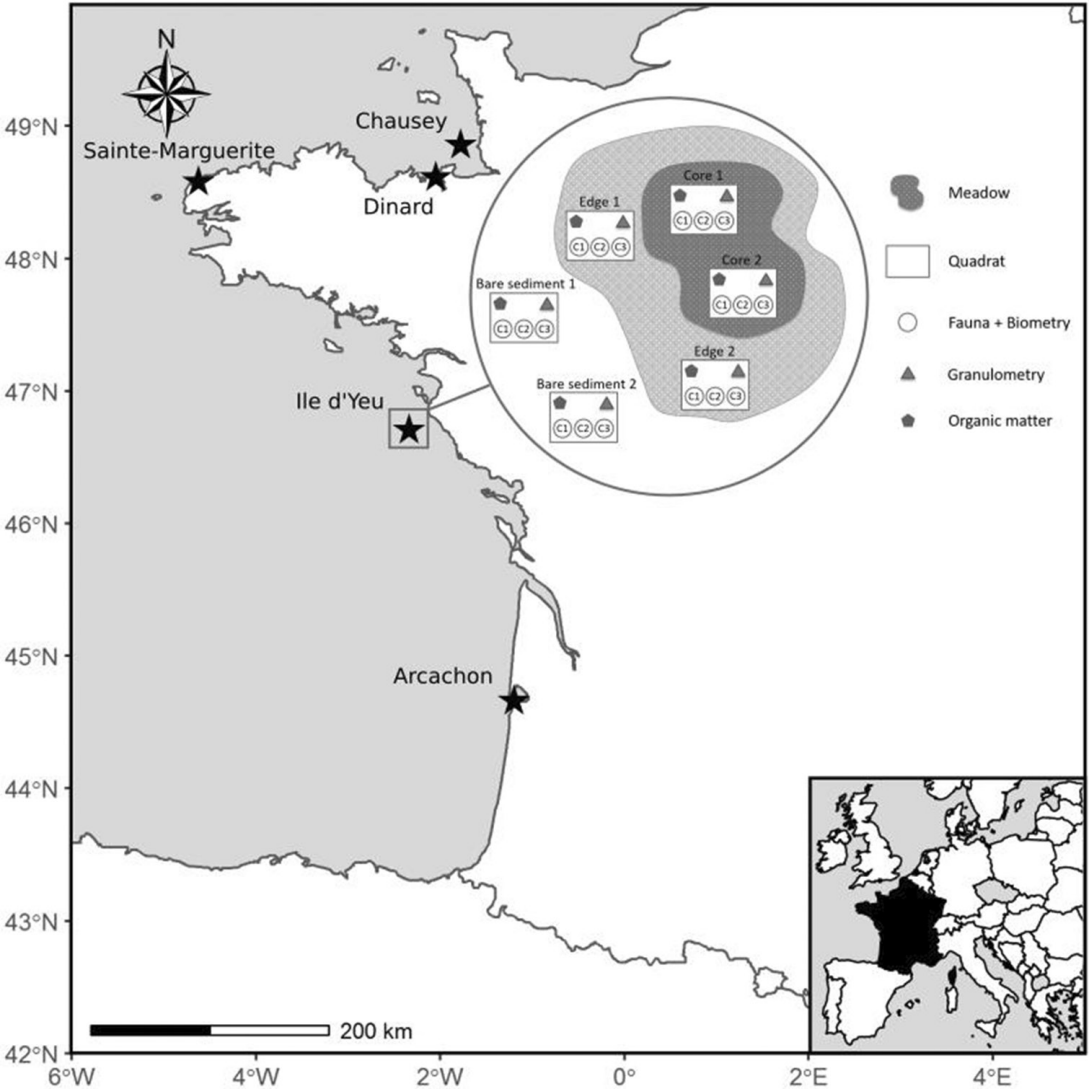
Map indicating the locations of the five study sites of *Zostera marina* meadows in France: three in the English Channel, and two in the Bay of Biscay. All sites were sampled in six different stations, that is, two in each habitat type (core, edge, and bare sediments).

Five sites along the coast of France were selected to quantify diversity in benthic macrofaunal assemblages associated with *Z. marina* meadows: three in the English Channel, and two in the Bay of Biscay (Figure 1). These sites were chosen to cover a range of environmental conditions in which *Z. marina* meadows can be found: from exposed, fully marine conditions (Ile d'Yeu and Chausey), to semi‐open habitats (Dinard and Sainte‐Marguerite; Boyé et al., 2017; Short, 2003), to sheltered bays with turbid waters (Arcachon). Sampling was carried out in autumn 2019 (late September to mid‐November) following a standardized protocol at each site. This sampling period corresponded to the season of maximum canopy development for eelgrass and the post‐recruitment period for most macroinvertebrate species (Grall, 2002; Moore & Short, 2006). To study community diversity and species composition associated with *Z. marina* meadows over short spatial scales, benthic macrofauna were sampled in three different habitat types at each sampling site. These habitat types were selected by using temporal mapping of the meadows based on field observations, acoustic mapping, and/or aerial photography of the meadows (Fournier et al., 2010; Rigouin et al., 2022; Rollet et al., 2011). The “core” of the meadow was characterized by perennial areas colonized by *Z. marina* for at least 10 years, the “edge” of the meadow was characterized by recently colonized (last few years) and temporally unstable eelgrass areas, and “bare sediments” were habitats not colonized by *Z. marina* (Figure 1, Figure S1).

In each site, two sampling stations at least 10 m apart were selected in each of three habitat types (core, edge, and bare sediment). At each sampling station, three samples of eelgrass shoots and sediments were collected to recover the associated fauna. These three samples were retrieved with three manual push cores (15 cm in internal diameter, to a depth of 15 cm; in other words 0.01 m^2^ per push core) that were pooled together (for a total of 0.03 m^2^ per sample). Care was taken to avoid damage to eelgrass leaves. The sediments and plants were placed into 1 mm nylon mesh collection bags, which allowed pre‐sieving and washing away of most sediments directly in the field (Figure 1). Once in the laboratory, the content of each sample was preserved in 70% ethanol. To ensure optimal species conservation, ethanol was replaced every 2 days, for a total of three renewals. In the laboratory, cores were sieved on a 1 mm mesh. Macrofauna was then extracted from the sediments and counted. All individuals belonging to polychaetes, gastropods, and bivalves were identified to the lowest taxonomic level possible, most often to the species level. These phyla were selected because they cover a broad diversity of traits and functions in the community, were the most abundant phyla within the samples, and were less likely to evade sampling compared with more vagile species. The sampling, thus, focused primarily on infaunal taxa but did not exclude epifaunal species living on *Zostera* leaves or on the sediment surface. All species names were used according to the World Register of Marine Species and references used for taxonomic identification are listed in Appendix S1 in Supporting Information. To ensure consistent taxonomic resolution across samples, a unique operator (A. Muller) was involved and uncertain identifications were cross‐checked by a taxonomic expert (G. Droual).

### 2.2 Morphometric measurements

All shoots in each sample were counted to measure *Z. marina* densities, for each of the two core and two edge sampling stations, in each of the five sites. For each sample, five shoots were randomly selected for morphometric measurements (i.e., a total of 15 measurements [five shoots per sample] for each station), which included sheath height, leaf length and width, and the number of leaves per shoot. Sheath height was measured from the first node to the leaf separation mark. The length of each leaf was measured from the node mark to the apex. The width was taken at mid‐length. The dry biomass of leaves, sheaths, roots, and rhizomes was measured separately, following 48 h of desiccation at 60°C. Total biomass and densities were expressed per square meter. To assess the relative investment of *Z. marina* between its above‐ground and below‐ground compartments, we calculated the ratio between the biomass of leaves and sheaths and the biomass of roots and rhizomes (Boyé et al., 2022). The leaf area index (LAI) was calculated as the average leaf surface area per shoot (leaf length times leaf width), multiplied by the shoot density. For all other variables (densities, sheath height, leaf length and width, number of leaves per shoot, proportion of broken leaves), mean values (and standard errors) were calculated. Broken leaves were retained in the calculation of average leaf length to reflect the physiological and mechanical impacts of the eelgrass environment (Boyé et al., 2022). However, leaves cleanly cut by the corer were removed to avoid bias related to the sampling method. All morphometric measurements are listed in Table S1.

### 2.3 Environmental variables

Two sediment cores were collected from each sampling station for measuring grain size distribution and organic matter content, respectively (Figure 1). Sediments were dried in an oven (72 h at 60°C) and separated into 25 fractions for which the weights were measured. Fractions were then grouped into gravels (>2 mm), sand (63–2 mm), and silt and clay (<63 μm; Fournier et al., 2012). Loss‐on‐ignition (450°C for 4 h) estimates of organic matter in sediments were conducted (Heiri et al., 2001).

Information regarding physical environmental conditions at each site (e.g., water temperatures, salinities, and current velocities) were obtained from the publicly available MARC database (https://marc.ifremer.fr/en), which modeled physical oceanographic parameters using the MARS3D hydrodynamic model (2.5 km resolution, 40 depth levels; https://marc.ifremer.fr/en). All variables were extracted daily for the year prior to the study at midday, at the deepest layer near the sediment surface. Given that the English Channel and the Bay of Biscay have different tidal regimes, from mega‐tidal in the central English Channel to meso‐tidal in the southern Bay of Biscay, tidal amplitude over a meadow varied depending on the geographical location of the sites. Tidal amplitudes used here were considered as the average annual difference in water level, calculated as the difference between the maximum and minimum water level predictions for each site based on the harmonic components of tidal heights and currents computed from the MARS3D models (https://marc.ifremer.fr/en; Lazure and Dumas, 2008) and the TidalToolBox (Allain, 2016). All environmental variables are listed in Table S1.

### 2.4 Biological traits

To assess functional diversity, biological traits were scored for polychaetes, gastropods, and bivalves, three phylogenetically diverse groups composed of a large diversity of species exhibiting a wide range of ecological strategies (Aldea et al., 2008; Gosling, 2015; Jumars et al., 2015; Teso et al., 2019). Eight biological traits were selected (Table S2), providing information related to the ecological functions performed by the associated macrofauna. These traits characterized the maximum size, feeding and reproductive ecology, mobility, and bioturbation potential of the species and were chosen to reflect key biological and ecological processes (Queirós et al., 2013; Solan et al., 2004; Thrush et al., 2006). Traits were all encoded as categorical variables with non‐mutually exclusive categories. Species were scored for each trait category based on their affinity using a fuzzy coding approach (Chevene et al., 1994), where multiple categories can be assigned to a species if appropriate, and allowed for incorporation of intraspecific variability in trait expression. A site‐by‐trait matrix containing information on the total abundance of each trait category by site was calculated using the matrix product of the site‐species matrix with the species‐trait matrix. Beforehand, the species scores within the species‐by‐trait matrix (fuzzy‐coded) were standardized to a sum of 1 (i.e., relative scores), to give the same weight to all traits, independently of the number of categories they were split into. Information for polychaetes was primarily extracted from Boyé et al. (2019), Fauchald & Jumars (1979), and Jumars et al. (2015). Information for gastropods and bivalves was obtained either from biological trait databases (www.marlin.ac.uk/biotic, www.univie.ac.at/arctictraits) or from publications (e.g., Bacouillard, 2019; Martini et al., 2020; Queirós et al., 2013; Thrush et al., 2006). Information was collected at the lowest possible taxonomic level and when missing was based on data available in other species of the genus, or in some cases, in the same family (only for traits with low variability for these families).

### 2.5 Statistical analyses

#### 2.5.1 α‐Diversity

Changes in total abundance, as well as taxonomic and functional diversity, were examined across different spatial scales ranging from within meadows (edge vs. core) to local habitat conditions (meadow vs. bare sediment) to regional habitat conditions (meadow vs. meadow) using three different α‐diversity measures. For comparisons among meadows as well as between meadows and bare sediments, samples collected in both core and edge habitats were used to characterize the meadow. The taxonomic diversity was estimated using the Simpson index applied to each sampled core. The Simpson diversity index was chosen because of its property of reducing the influence of rare species (Hill, 1973), to emphasize the effect of species accounting for most of the total abundance. The functional structure of benthic assemblages was characterized using two complementary indices: functional richness (FRic) and functional evenness (FEve, Laliberté & Legendre, 2010; Mouchet et al., 2010; Villéger et al., 2008). For that purpose, a trait‐space was built using a principal coordinate analysis (PCoA) based on Euclidean distances applied to the normalized fuzzy coded species‐trait matrix (Boyé et al., 2019). Five axes were retained to characterize the trait space (Boyé et al., 2019; Mouillot et al., 2021) which represented 30% of the total variance of the species‐trait matrix (*R* ^2^‐like ratio; Laliberté et al., 2014). Hence, assemblages with less than five species are not considered in this analysis (Boyé et al., 2019). For both taxonomic and functional diversity, two‐way nested ANOVAs were used to test for differences between habitat types and sites (habitat type nested within site) with habitat type defined in a first analysis as (i) core versus edge, and in a second as (ii) meadow versus bare sediment (considering both edge and core samples in the meadows). Moreover, one‐way ANOVA was used to test for differences among meadows (differences across localities considering both core and edge samples). Pairwise comparisons were then carried out using Tukey‐tests (*p* < .05).

#### 2.5.2 β‐Diversity

Variation in community composition within *Z. marina* meadows (i.e., core and edge) across the five sites was visualized using a principal component analysis (PCA) performed on Hellinger‐transformed species abundances collected in each sampling unit (i.e., sediment cores). Abundance‐based dissimilarities can be strongly influenced by overabundant species or by a high proportion of rare species. Applying a Hellinger transformation to abundance data allows Euclidean‐based methods to be used, while not overweighting rare species (Legendre & Gallagher, 2001).

To understand differences among communities, taxonomic and functional β‐diversity were assessed using pairwise Jaccard dissimilarity (Jaccard, 1912) and its two components: nestedness and turnover (Baselga, 2017; Villéger et al., 2013). Taxonomic β‐diversity was computed by first transforming abundances in each sample into presence‐absence. Functional β‐diversity was quantified and decomposed using convex‐hulls computed using a trait space built from the two first axes of a fuzzy correspondence analysis (Villéger et al., 2013).

### 2.6 Determinants of variation in diversity

Piecewise structural equation models (pSEM; Lefcheck, 2016) were used to explore the direct and indirect effects (through eelgrass morphometric responses) of environmental factors on benthic communities. SEM is a powerful, multivariate technique that is increasingly used to test and evaluate multivariate causal relationships. Specifically, SEM allows testing the direct and indirect effects on pre‐assumed causal relationships, ultimately facilitating the identification of cascading effects (Lefcheck et al., 2015). We implemented an SEM that considered the effects of environmental and morphometric variables on taxonomic and functional α‐diversity as well as taxonomic β‐diversity. α‐diversity was represented by total abundance, species richness, FRic, whereas β‐diversity was represented as the first two axes of the PCA performed on Hellinger‐transformed species abundances. In this analysis, only communities sampled in the cores and edges of meadows were considered (bare sediment communities were excluded).

To reduce the number of parameters considered in the pSEM and select the most relevant biotic (morphometric measurements of *Z. marina*) and abiotic predictors (environmental variables), collinear variables were removed using a variance inflation factor analysis with a threshold of ten (Figure S5; Legendre & Legendre, 2012). This led to the removal of 7 variables (see Table S2). A redundancy analysis was performed between the Hellinger‐transformed abundances and the remaining predictors (biotic and abiotic) using a stepwise selection procedure based on adjusted coefficients ($R_{\mathrm{adj}}^{2}$; Figure S6; Blanchet et al., 2008). This resulted in the selection of three environmental and three morphometric variables: temperature, current velocity, tidal amplitude, below‐ground biomass, leaf width, and leaf length.

Using the selected variables, a saturated pSEM containing 52 paths was built. This pSEM contains both directed relationships, assumed from the literature and expert knowledge, but also includes correlated errors where the link between variables is accounted for with no assumption regarding causality. The model assumed a Gaussian error structure for all explained variables. Model quality was assessed using *R* ^2^ and Fisher's *C* statistics (Lefcheck, 2016). From this model, all non‐significant paths were removed (Garrido et al., 2022). We then refitted the model, only keeping the significant paths (*n* = 30) to refine coefficient estimates (Table S3).

All statistical analyses were performed in R 4.0.3 (R Development Core Team, October 2020) using the packages G2Sd (Fournier et al., 2014), ade4 (Dray & Dufour, 2007), vegan (Oksanen et al., 2022), FD (Laliberté et al., 2014), betapart (Baselga & Orne, 2012), and piecewiseSEM (Lefcheck, 2016).

## 3 RESULTS

A total of 90 community samples were collected across the five sites, for a total of 9277 individuals and 138 species (33 bivalves, 20 gastropods, and 82 polychaetes). Rare species accounted for a large proportion of the samples: 43% of the species were observed in a single sample and 38% were represented by one or two individuals.

### 3.1 Meadows versus bare sediments

The abundance and diversity of species differed between meadows (core + edge) and bare sediments across the different sites (Table 1, Figure S1). Specifically, average abundance (ind. m^−2^) and species richness were significantly higher in meadows than in bare sediments at all sites, except Ile d'Yeu (Table 1). Communities associated with meadows showed less variation in both average abundance (2384–6188 ind. m^−2^) and species richness (11–19), than bare sediments where abundances varied from 303 to 2729 ind. m^−2^ while species richness varied from 4 to 11 (Table 1). Similarly, when considering functional diversity indices, benthic communities in bare sediments were characterized by a small functional space (low FRic) with evenly distributed trait abundances (high FEve). In comparison, communities associated with meadows had larger functional spaces (higher FRic) with abundances being concentrated on a few trait combinations (low FEve; Table 1). This indicates that the dominant species tended to share the same functional traits and that a large part of the functional space was occupied by less abundant species with rarer trait combinations.

**TABLE 1:** Spatial variability in α‐diversity indices (species richness, Simpson's index, and abundance per m^2^) for benthic assemblages associated to *Zostera marina* meadows at five sites located in metropolitan France. Mean values are displayed with their associated standard deviations. Values in bold indicate the number of species unique to a given habitat type within the site. Different letters/numbers (lowercase letters for core vs. edge, capital letters for bare sediment vs. meadow, and numbers for meadow vs. meadow) indicate significant differences at Tukey's test (*p* < .05).

Sites	Habitat types	Total species richness	Mean species richness	Mean simpson diversity	Mean abundance per m^2^	Mean FRic	Mean FEve
Chausey	Bare sediment	13–**5 (7%)**	4 ± 4^A^	0.50 ± 0.3^A^	322 ± 527^A^	0.13 ± 0.19^A^	0.62 ± 0.1
	Meadow	65–**57 (81%)**	17 ± 4^B,2^	0.83 ± 0.04^B^	2483 ± 838^B,1^	0.59 ± 0.13^B,2^	0.72 ± 0.05^13^
	Core	41–**15 (21%)**	15 ± 2	0.84 ± 0.03	2384 ± 836	0.58 ± 0.15	0.71 ± 0.04
	Edge	50–**23 (32%)**	19 ± 4	0.83 ± 0.1	2582 ± 906	0.60 ± 0.11	0.72 ± 0.05
Dinard	Bare sediment	31–**11 (19%)**	11 ± 8^A^	0.67 ± 0.35	1004 ± 843^A^	0.38 ± 0.23^A^	0.75 ± 0.07^A^
	Meadow	46–**26 (46%)**	16 ± 4^B,2^	0.69 ± 0.9	5835 ± 1577^B,1^	0.61 ± 0.12^B,2^	0.62 ± 0.06^B,23^
	Core	32–**6 (10%)**	18 ± 2	0.74 ± 0.04	6189 ± 898	0.63 ± 0.08	0.65 ± 0.05
	Edge	40–**11 (19%)**	14 ± 5	0.64 ± 0.1	5482 ± 2089	0.60 ± 0.15	0.58 ± 0.05
Sainte marguerite	Bare sediment	22–**11 (23%)**	8 ± 2^A^	0.53 ± 0.17^A^	2729 ± 2105^A^	0.28 ± 0.04^A^	0.60 ± 0.16
	Meadow	37–**26 (54%)**	13 ± 3^B,2^	0.71 ± 0.14^B^	4550 ± 2038^B,1^	0.46 ± 0.18^B,2^	0.61 ± 0.11^2^
	Core	28–**8 (17%)**	14 ± 2	0.65 ± 0.15	5763 ± 976	0.51 ± 0.19	0.59 ± 0.03
	Edge	28–**9 (19%)**	11 ± 2	0.76 ± 0.12	3338 ± 2159	0.40 ± 0.16	0.62 ± 0.15
Ile d'Yeu	Bare sediment	8–**1 (3%)**	4 ± 2^A^	0.55 ± 0.30	429 ± 231	0.04 ± 0.04	0.77 ± 0.16
	Meadow	38–**31 (82%)**	8 ± 4^B,1^	0.69 ± 0.18	774 ± 449^2^	0.22 ± 0.18^1^	0.73 ± 0.14^1^
	Core	34–**22 (57%)**	11 ± 4^a^	0.78 ± 0.11^a^	1010 ± 526	0.32 ± 0.20^a^	0.75 ± 0.08^a^
	Edge	16–**3 (8%)**	5 ± 1^b^	0.59 ± 0.2^b^	537 ± 181	0.12 ± 0.10^b^	0.72 ± 0.19
Arcachon	Bare sediment	19–**5 (10%)**	6 ± 2^A^	0.79 ± 0.5	303 ± 154^A^	0.24 ± 0.2^A^	0.84 ± 0.07^A^
	Meadow	43–**29 (60%)**	14 ± 3^B,2^	0.70 ± 0.15	5505 ± 3787^B,1^	0.65 ± 0.08^B,2^	0.63 ± 0.08^B,123^
	Core	32–**8 (17%)**	13 ± 2	0.64 ± 0.2	5949 ± 4305	0.56 ± 0.1	0.61 ± 0.08
	Edge	34–**7 (15%)**	15 ± 3	0.75 ± 0.1	5061 ± 3541	0.56 ± 0.1	0.66 ± 0.09

### 3.2 Variation within and across eelgrass meadows

#### 3.2.1 Patterns of α‐diversity

**FIGURE 2:**
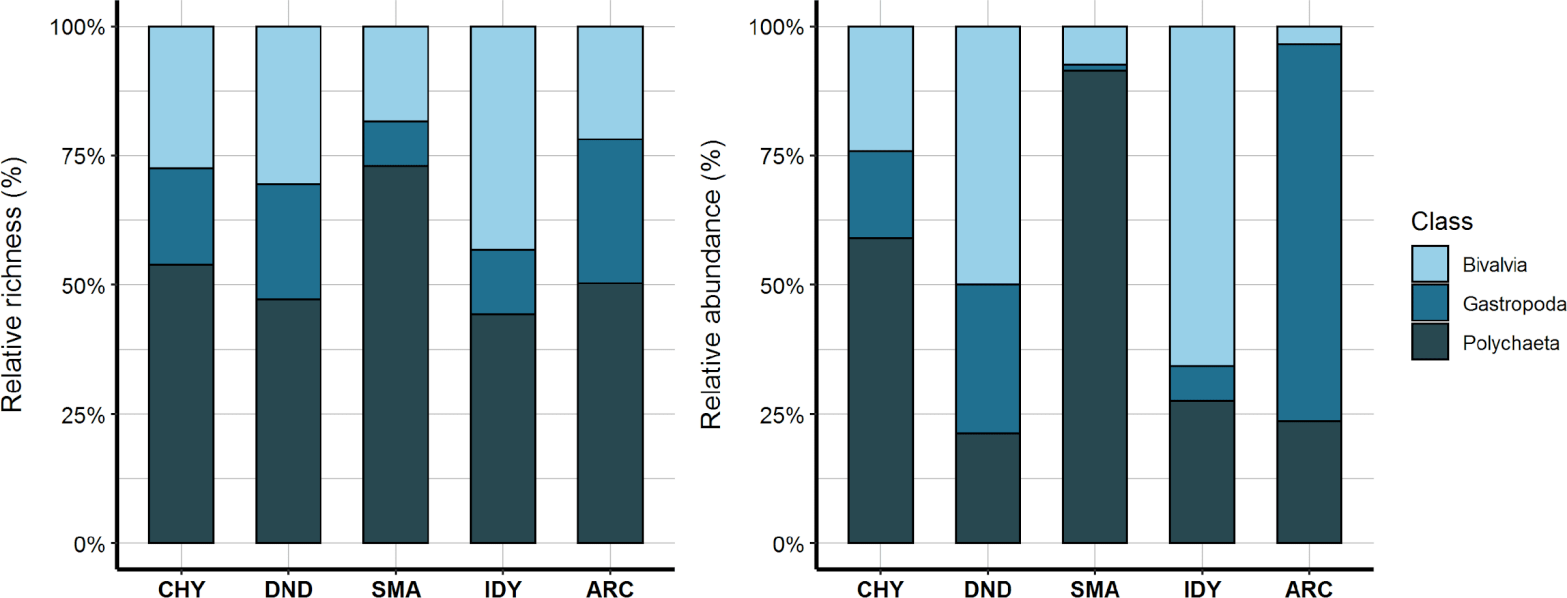
Richness and relative abundance of the different taxonomic groups present at each of the five sites: Chausey (CHY), Dinard (DND), Sainte‐Marguerite (SMA), Ile d'Yeu (IDY), and Arcachon (ARC).

In contrast to average species richness and Simpson index, which displayed comparable values among meadows, marked spatial differences were observed for average abundances (Table 1). For instance, macrofaunal abundance was sevenfold greater in the most densely populated meadow (Dinard) relative to the most sparsely populated meadow (Ile d'Yeu). In particular, striking differences were observed in abundances of polychaetes, gastropods, and bivalves among sites, with Chausey, Dinard, and Ile d'Yeu presenting higher abundances of bivalves whereas Arcachon presented a higher abundance of gastropods, and Sainte‐Marguerite of polychaetes (Figure 2; Figure S2). Nevertheless, six taxa were found in all meadows: the bivalves *Loripes orbiculatus*, *Lucinoma borealis*, and *Parvicardium scabrum*, the gastropod *Tritia reticulata* and the polychaetes *Euclymene* sp. and *Melinna palmata* (Figure S3).

Regarding habitats within meadows, no significant differences were observed in abundance, richness, or Simpson diversity between core and edge habitats except for Ile d'Yeu, where core habitats had significantly higher values of richness and Simpson diversity than edge habitats.

#### 3.2.2 Variation in taxonomic composition (taxonomic β‐diversity)

**FIGURE 3:**
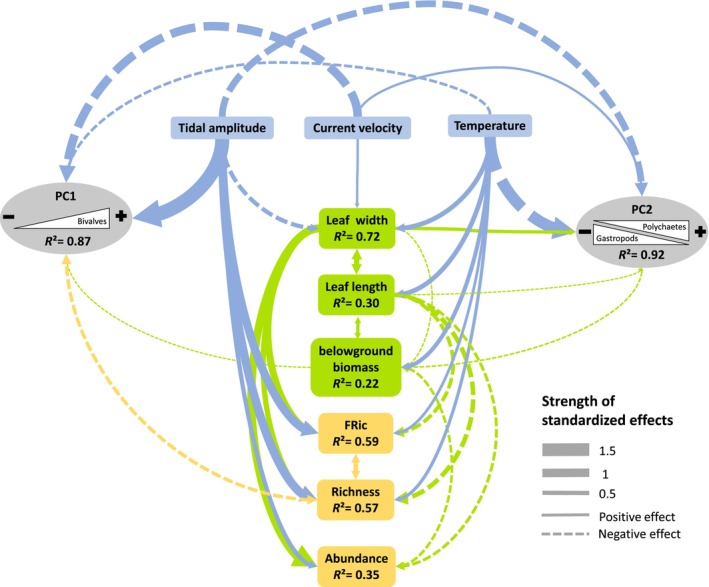
Principal component analyses of the Hellinger‐transformed abundance of taxa associated with the five *Zostera marina* beds sampled on two habitat types (core and edge). (a) The sites for each point sampled in core and edge with their 95% confidence dispersion ellipses, represented in scaling 1 (distance biplot) preserving the distances among the sites. Within‐site dispersions represent variation of the communities among habitat types. (b) Positions of the species for which the two first axes represented at least 40% (cumulative *R* ^2^) of the variance, represented in scaling 2 (correlation biplot) preserving the covariances among the species.

Assemblage composition associated with both core and edge habitats showed strong differences among meadows (Figure 3a; Figure S4). The first axis of the PCA performed on Hellinger transformed abundances (PCA_Abundance_; 20.5% of total variability) discriminated samples (from 5 sites × 2 habitat types × 2 sampling stations × 3 cores) based on bivalve composition, with the Dinard meadow showing the highest diversity of bivalves and the greatest abundances in species such as *Loripes articulatus, Lucinoma borealis*, and *Tricolia pullus*. The second axis of PCA_Abundance_ (16.6% of total variability) discriminated samples based on gastropod and polychaete compositions, with the Sainte‐Marguerite meadow exhibiting the highest abundances of polychaetes including *Platynereis dumerilii* and *Spio* cf. *martinensis* whereas the Arcachon meadow exhibited the highest diversity and abundances of gastropods, including *Jujubinus striatus* and *Bittium reticulatum* (Figure 3b; Figure S4).

#### 3.2.3 Variation in functional composition (functional β‐diversity)

**FIGURE 4:**
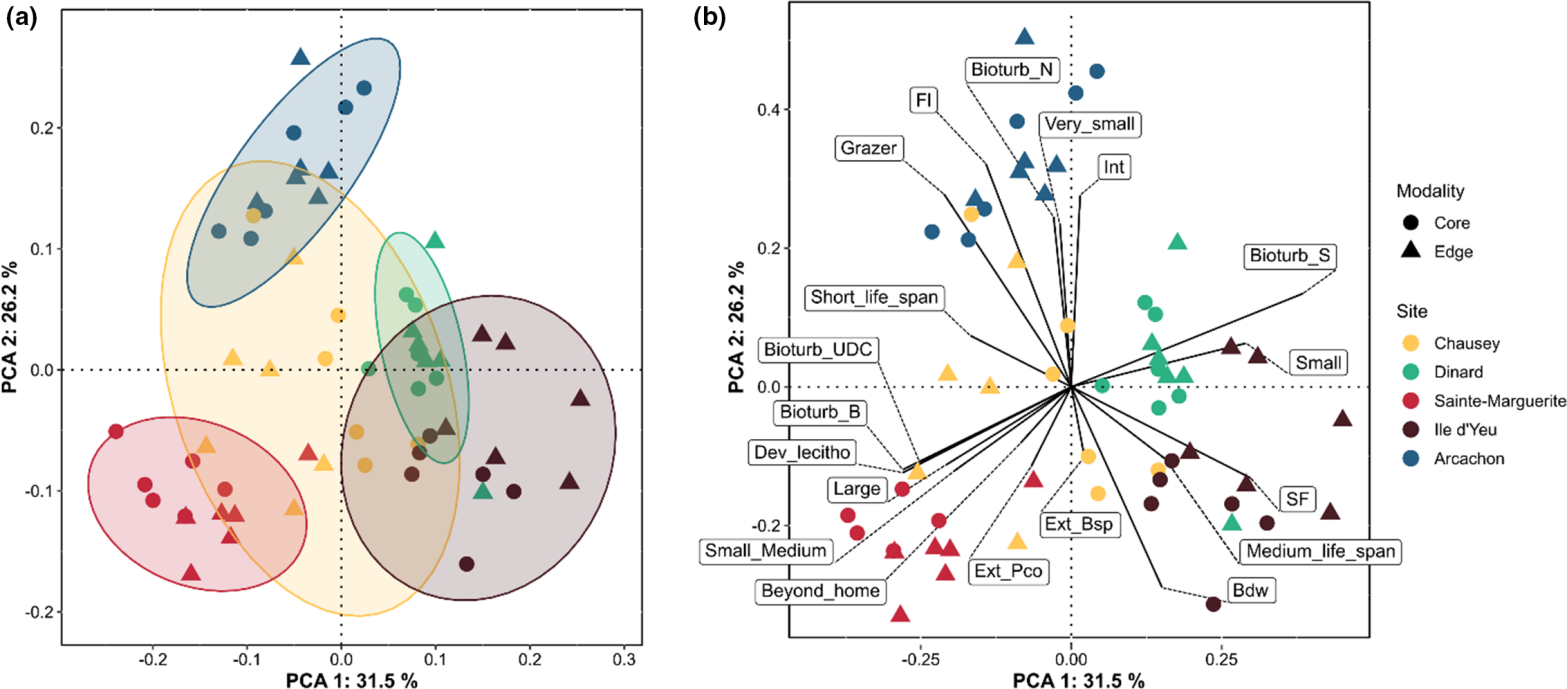
Principal component analysis of Hellinger‐transformed abundances of trait categories. (a) The sites for each point sampled in core and edge with their 95% confidence dispersion ellipses, represented in scaling 1 preserving the distances among the sites. (b) The positions of trait categories for which the two first axes represented at least 40% (cumulative *R* ^2^) of the variance, represented in scaling 2.  Trait abbreviations: Bdw, Burrow dwelling, Bioturb_B, Bioturbation biodiffusors, Bioturb_N, Bioturbation none, Bioturb_S, Bioturbation surficial modifiers, Bioturb_UDC, Bioturbation upward/downward conveyors, Dev_lecitho, Development lecithotrophic, Ext_Bsp, External broadcast spawner, Ext_Pco, External pseudocopulation, Fl, Free living, Int, Internal, SF, Suspension feeder.

The PCA performed on trait category abundances (PCA_trait_) gave a complementary vision to that of the PCA_Abundance_. The first axis of PCA_trait_ discriminated assemblages characterized by the abundance of small suspension feeders and surficial modifiers with medium life span (positive values; characteristic of Dinard and Ile d'Yeu) from assemblages characterized by a greater abundance of large biodiffusers and upward/downward conveyors with short life spans (negative values; characteristic of Sainte‐Marguerite). The second axis of PCA_trait_ represented a gradient in the abundance of very small free‐living grazers with little effect on bioturbation (greater abundance for positive values), typical of the Arcachon meadow (Figure 4).

#### 3.2.4 Decomposing taxonomic and functional β‐diversity into nestedness and turnover

**FIGURE 5:**
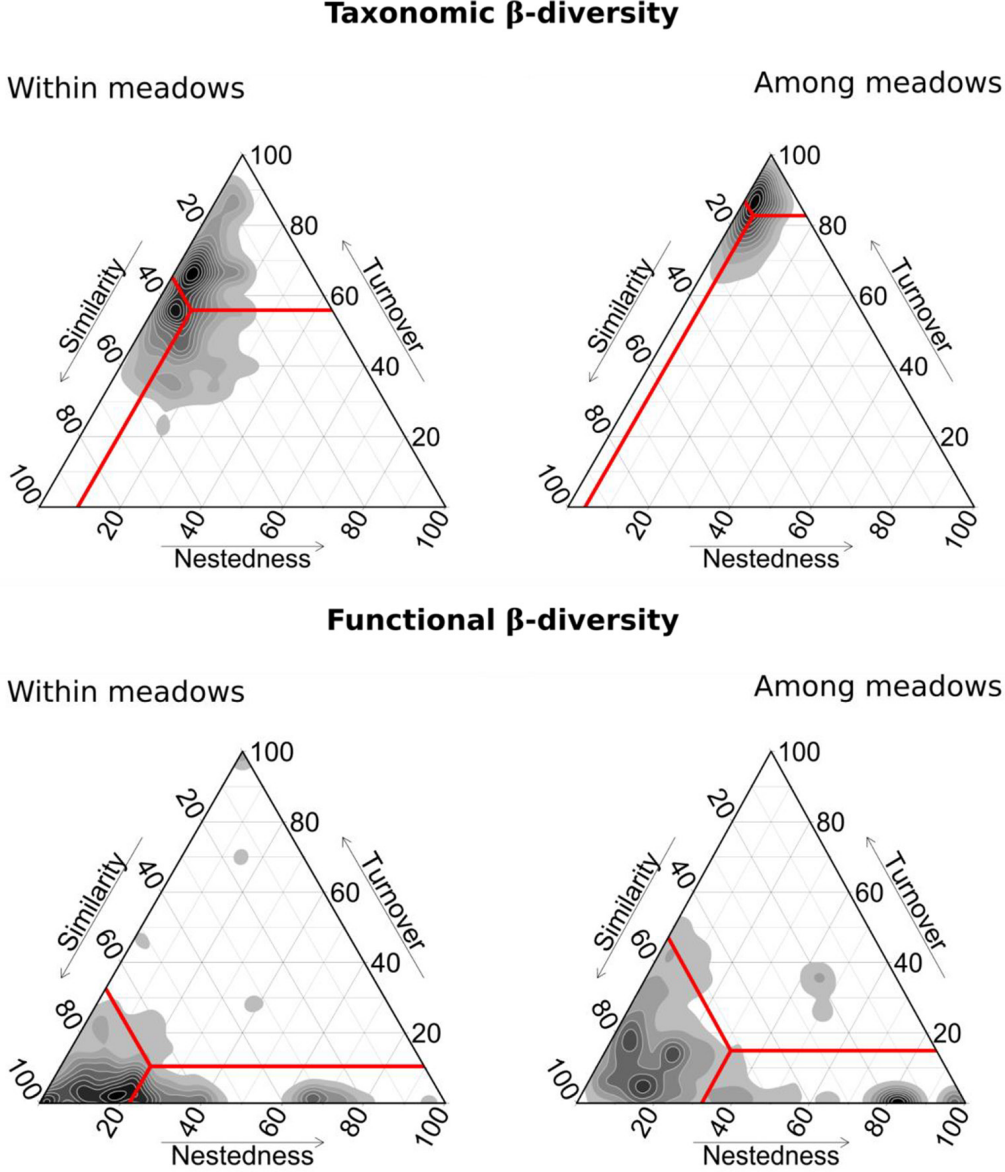
Triangular plots illustrating the spatial variation in taxonomic β‐diversity. Jaccard dissimilarity between the species composition (presence/absence data) of the five seagrass beds was used to quantify their similarity, and the two components of β‐diversity, nestedness (i.e., influenced by the difference in the number of species between the two communities) and turnover (i.e., species replacement between two communities). Contributions were calculated separately for comparisons between pairs of samples belonging: to the same meadow (within meadows), to pairs of samples from different meadows (among meadows). Red lines indicate the centroid value for each graph with its associated mean values for the three components of dissimilarity.

Regarding taxonomic β‐diversity, assemblages within meadows were always more similar (34.7 ± 12.8%) than assemblages among meadows (13.0 ± 8.1%). The turnover component accounted for most variation in taxonomic β‐diversity (56% within meadows and 87% among meadows; Figure 5) while nestedness only had a marginal influence (10% within meadows and 4% among meadows; Figure 5). Similar results were obtained for functional β‐diversity with greater similarity within meadows (67 ± 27%; Figure 5) than among meadows (53 ± 27%; Figure 5). These high similarity levels indicate high degrees of overlap in the functional space of the different assemblages both among and within meadows. Functional β‐diversity was mostly driven by nestedness (22 ± 23% within meadows and 32 ± 30% among meadows; Figure 5) rather than by turnover (10 ± 20% within meadows and 15 ± 18% among meadows; Figure 5).

#### 3.2.5 Variation in community structure in relation to environmental conditions and morphological characteristics of the meadows

**FIGURE 6:**
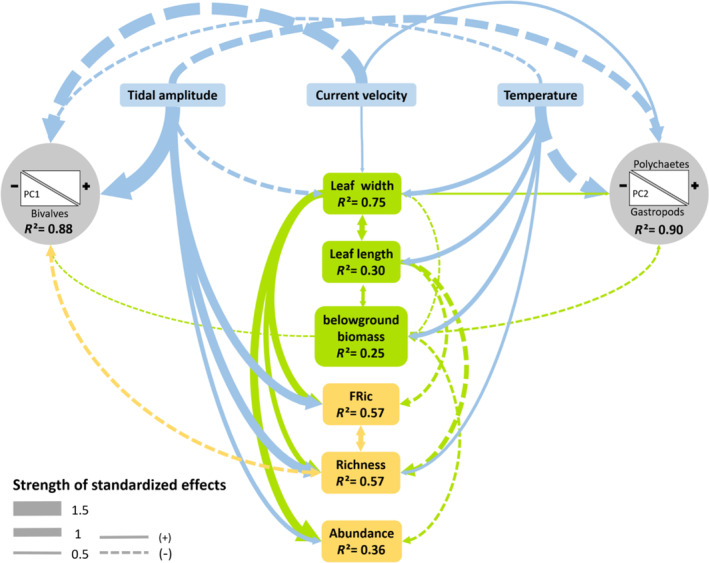
Best model fitted with piecewiseSEM (structural equation modeling) describing the relationships among *Zostera marina* traits (in green), environmental variables (in blue), and taxonomic and functional diversity measures (α‐ and β‐diversity). The *R* ^2^ values in the boxes (i.e., response variables) represent the total variance explained by all the predictors related to that box. Arrows indicate directional effects. Double‐headed arrows indicate correlated errors. Correlated errors represent unmeasured sources of variance that are influencing the relationship between two variables, acknowledging the correlation between the two variables without imparting a causal relationship. Arrows with solid and dot lines indicate positive and negative relationships, respectively. Line thickness is proportional to the standardized effect size (regression coefficient) of each relationship (Table S3).

Overall, the pSEM fitted the data well (AIC = 123.25, *χ* ^2^ = 33.25, *p* = 0.60, Figure 6). Regarding β‐diversity patterns (regional scale), tidal amplitude had the greatest effects on spatial variation in assemblage composition, having direct effects on the abundance and diversity of bivalves (positive correlation with the first axis of PCA_Abundance_, *β* = −1.66, *p* < .001) and gastropods (positive correlation with the second axis of PCA_Abundance_, *β* = −0.79, *p* < .001). Assemblage composition also varied with temperature and current velocity, with direct correlations found on both bivalve (First axis of PCA_Abundance_, *β* = −0.32, *p* < .001 and 1.00, *p* < .001, respectively) and gastropod (second axis of PCA_Abundance_, *β* = −1.37, *p* < .001 and *β* = 0.30, *p* < .001, respectively) abundances. Direct effects of plant traits on assemblage composition were small compared with the effects of environmental variables, with below‐ground biomass having positive effects on bivalve abundances (negative correlation with the first axis of PCA_Abundance_, *β* = −0.14, *p* < .05) and negative effects on the proportion of polychaetes (negative correlation with the second axis of PCA_Abundance_, *β* = −0.18, *p* < .01). Indirect effects of environmental variables were also observed on assemblage compositions with temperature having an indirect effect on bivalve abundance through its effect on below‐ground biomass.

Regarding α‐diversity patterns (local scale), taxonomic and functional richness were affected by both environment and eelgrass traits, with tidal amplitude having the greatest effect (*β* = 1.00, *p* < .001 and *β* = 0.97, *p* < .001, respectively), followed by leaf width (*β* = 0.62, *p* < .01 and 0.82, *p* < .001, respectively), leaf length (*β* = −0.53, *p* < .001 and *β* = −0.42, *p* < .01, respectively), and temperature (*β* = 0.37, *p* = .05 and *β* = .32, *p* < .05, respectively). Species abundances were influenced by leaf width, below‐ground biomass, and tidal amplitude (*β* = 0.96, *p* < .001, *β* = −0.30, *p* = .05, and *β* = 0.50, *p* < .01, respectively). Indirect effects of environmental variables were also observed, with temperature, tidal amplitude, and current velocity all having indirect effects on species abundances, as well as taxonomic and functional richness, mediated by their effects on eelgrass traits (leaf width and length; Figure 6). Taxonomic richness was also correlated with changes in assemblage composition (direct negative correlation with the first axis of PCA_Abundance_, *β* = −0.39, *p* < .01). Given that taxonomic richness is influenced by both environment and plant traits, retroactive effects of plant traits also played a role in regional differences in assemblage composition. All coefficients and their associated *p*‐values for the pSEM are presented in Table S3.

## 4 DISCUSSION

Eelgrass supports highly productive habitats that have been shown not only to enhance community diversity and biomass but also to affect key ecological functions such as primary and secondary production (Boyé et al., 2019; Duffy, 2006; Heck et al., 2008). Here, multiple facets of biodiversity were examined in five meadows spanning ~800 km of the Atlantic coast of France to better understand the factors that explain community structure associated with eelgrass at different spatial scales.

### 4.1 Local variation in diversity

At the local scale, both species richness and abundances were significantly greater in meadows than in bare sediments in nearly all sites. On average, <15% of the species were found only in the bare sediments, while more than 60% were unique to meadows. Eelgrass has been shown to favor high levels of species richness and densities (Edgar et al., 1994; Fonseca et al., 1990; Fredriksen et al., 2010; Orth et al., 1984; Stoner, 1980; Törnroos et al., 2013) likely due to higher availability of trophic resources (Duffy, 2006; Hemminga & Duarte, 2000) and enhanced shelter provisioning combined with lower predation (Heck & Orth, 2006). The results confirm that eelgrass meadows support greater species richness and abundance than geogenic habitats (Boyé et al., 2019; Henseler et al., 2019), and are thus of high conservation value (Whippo et al., 2018).

Benthic assemblages found in the core or edges of the meadows did not show strong differences in richness or abundance, although the core of some meadows tended to have greater diversity or richness (but none showed both). Studies that examined fine‐scale differences in diversity within meadows did not identify any consistent trend when comparing core and edge sectors, with most taxa showing no significant edge responses (Boström et al., 2011). However, mobile species such as crustaceans and fish have shown greater densities along the edges of meadows (Boström et al., 2006; Boström et al., 2011). Taxa with high mobility may respond differently to habitat edges than infaunal species. The absence of highly mobile species in our dataset may therefore partly explain why no differences in abundance or richness were detected between core and edge habitats within the meadows. In sum, at the local scale, community diversity and abundance were strongly favored by the presence of meadows over bare sediment, but habitat types within meadows did not have a strong effect on the assemblages studied, suggesting that core or edge patches of eelgrass may provide similar benefits to benthic biodiversity.

### 4.2 Regional variation in assemblage composition

Spatial variation was observed in assemblages across the five meadows both from a taxonomic and functional perspective. Taxonomic differences among meadows were accompanied by changes in the abundance of specific trait combinations. The Dinard meadow was rich in bivalves and characterized by high abundances of small suspension feeders, the Sainte‐Marguerite meadow by greater abundances of small to large biodiffusers and upward/downward conveyors, most of which were polychaetes, and the Arcachon meadow by greater abundances of small free‐living grazers with little effect on bioturbation (gastropods). The Chausey meadow was composed of a combination of all the traits found in the other meadows. Previous work on eelgrass diversity has also shown significant variation in species composition among meadows (Henseler et al., 2019; Törnroos et al., 2013; Wong & Dowd, 2015).

In the analysis of Jaccard dissimilarity, high taxonomic turnover was observed among meadows, with taxonomic turnover being often >70%, while functional turnover only reached 10% on average. Low functional turnover coupled with low functional evenness indicates that communities associated with eelgrass are largely dominated by a limited set of traits. Environmental filtering associated with the meadows (i.e., habitat filtering) has led to communities rich in small suspension feeders and surficial modifiers, large biodiffusers and upward/downward conveyors, and small free‐living grazers. Habitat filtering has also been inferred for other eelgrass meadows in France (Boyé et al., 2019; Ouisse et al., 2012). Nevertheless, high taxonomic turnover was observed among meadows, with many rare species and rare traits being limited to a given meadow. These observations indicate the presence of a large number of transient species both spatially (species observed in one meadow but not another) or temporally (species present only during a given period) in eelgrass meadows, in agreement with previous work (Boyé et al., 2019; Umaña et al., 2017). This high turnover, coupled with stable richness is typical of meta‐communities experiencing source‐sink dynamics (Hillebrand et al., 2008; Leibold et al., 2004). At the community level, source habitats have high abundances of a given set of species, while sink habitats have low abundances of species that use the meadow temporarily to reproduce, feed or escape from predators (Boström & Bonsdorff, 2000; Bouma et al., 2009). Given that eelgrass has a high turnover of rare species and traits, meadows may serve as a sink habitat for a large number of species that spend much of their life cycle elsewhere.

Despite strong differences in community composition among meadows, species nestedness did not vary significantly, with richness remaining comparable from meadow to meadow. Such a narrow range of variation in species richness may indicate that the studied meadows are at their carrying capacity (*sensu* Hansen et al., 2011; Boyé et al., 2017).

### 4.3 The role of biotic and abiotic factors in shaping communities

Environmental factors such as temperature, salinity, and tidal amplitude have been shown to affect community structure associated with eelgrass (Boström & Bonsdorff, 1997, 2000; Bowden et al., 2001). At local scales, several meadow characteristics (e.g., biomass, LAI, shoot density) have also been shown to directly influence species‐level responses (e.g., growth, mortality, predation, movement, reproduction; Fonseca & Bell, 1998; Heck & Orth, 2006; Koch & Verduin, 2001; Robbins & Bell, 2000). However, the relative importance of biotic and abiotic factors in explaining variation in community composition has proven more difficult to understand because these factors typically covary (Bowden et al., 2001; Hovel et al., 2002; Turner et al., 1999). In line with studies conducted on other foundation species (Lamy et al., 2020; Miller et al., 2018), pSEM was used here to clarify the relative contributions of biotic and abiotic factors on the taxonomic and functional structure of assemblages associated with eelgrass. Specifically, the pSEM showed that spatial variation in assemblage composition among meadows is primarily explained by direct effects of the environment. For instance, temperature and tidal amplitude had large effects on the abundance and prevalence of bivalves and gastropods in the Dinard and Arcachon meadows. In contrast, current velocity was associated with assemblages dominated by polychaetes in Sainte‐Marguerite or Ile d'Yeu, the latter hosting species specific to certain types of sediment (e.g., genus *Magelona*). Overall, plant traits had a minor role in explaining spatial variation in community composition at the regional scale.

Beyond its effect on assemblage composition, the environment was also found to affect eelgrass traits, which in turn affected local community richness and abundance (i.e., cascading effects; Barnes et al., 2017). Temperature favored higher leaf width, length, and below‐ground biomass, leading to positive indirect effects that amplified its already positive direct effects on community diversity. On the other hand, tidal amplitudes had negative effects on leaf width, leading to negative indirect effects on richness that partially counterbalanced the positive direct effects observed. Such contradictory trends in the paths of direct and indirect effects are not unusual in natural ecosystems (e.g., Barnes et al., 2017) and highlight that the net effect of environmental changes on benthic diversity cannot be fully apprehended without a thorough understanding of the complex mediating role of foundation species (Bulleri et al., 2016; Harley et al., 2006).

In contrast to previous studies, the pSEM did not demonstrate a correlation between low tidal amplitude and canopy height (Larkum et al., 2006), or between wave exposure and below‐ground biomass (Fonseca & Bell, 1998). The absence of these expected correlations is possibly the result of the temporal dynamics of meadows. Strong inter‐annual variability has been reported within meadows in several sites on the Atlantic coast of France, including some of the same sites studied here (Boyé et al., 2022). Our study considered a single season of a particular year, which may not have captured all of the possible environmental drivers that influence eelgrass morphology.

While community composition was primarily driven by spatial variation in environmental conditions at the regional scale, local measures of diversity, including species abundances as well as taxonomic and functional richness were affected more or less equally by the environment and plant traits. Plant traits have previously been associated with community diversity in eelgrass. For example, community diversity has been observed to be positively correlated with the above‐ground structure of eelgrass (Attrill et al., 2000; Leopardas et al., 2014). Similarly, greater leaf area was found to favor the abundance of mesograzers (Fredriksen et al., 2005) or species living directly on *Zostera* leaves, such as *P. dumerilii* (Jacobs & Pierson, 1979). Leaf area has also been correlated with the presence of species presumed to use the eelgrass bed as a foraging and spawning site from adjacent habitats (e.g., *Pusillina inconspicua* or *Musculus costulatus*; Rueda et al., 2008).

One variable that may not have been adequately quantified in the pSEM relates to the availability of trophic resources in eelgrass beds. Detrital material can be an important resource in eelgrass, favoring diversity (Bologna & Heck, 1999). Here, the organic matter present in the sediments was quantified as a proxy of detrital trophic resources but explained little of the variation in the assemblages studied. The accumulation of drifting algae as is commonly observed in meadows such as Sainte Marguerite (Boyé et al., 2019), could explain why this meadow was particularly rich in polychaetes, such as *Spio* cf. *martinensis*, but the variables quantified in the study did not allow verification of this hypothesis. Future studies may also consider quantifying epiphytic or detrital biomass directly, to better understand how these two components may influence eelgrass biodiversity.

Overall, the pSEM made it possible to quantify the relative contributions of environmental conditions and meadow characteristics on local and regional diversity associated with eelgrass communities. Environment has a strong effect at the regional scale, while locally, diversity was affected by both environment and plant traits. Since eelgrass traits were also directly influenced by the environment, our analysis indicates that the environment also exerts a local control on eelgrass‐associated assemblages. These indirect effects highlight the complex nature of eelgrass ecosystems and the potentially cascading effects the environment can have on eelgrass communities.

### 4.4 Conservation and management action

The results presented here have implications for the future conservation of eelgrass meadows. By showing that long‐standing, stable habitats (meadow cores) and more recently colonized, presumably unstable habitats (meadow edges), harbor similar communities, our results indicate that both habitats provide similar benefits to invertebrate biodiversity. Once eelgrass becomes established in a new area, the associated fauna reaches abundances and levels of diversity comparable with long‐standing stable cores, at least for infaunal and moderately mobile taxa. Eelgrass meadows are known to expand or recede over annual or pluriannual cycles, but regressions may not be a cause for concern in terms of biodiversity. Protection of eelgrass, independent of age, density, or stability, is likely to ensure a number of ecological functions.

The results further suggest that meadow traits such as density and aboveground biomass are only weakly correlated with the diversity of the associated fauna, and may not provide good proxies for diversity. Furthermore, morphological traits of eelgrass are tightly linked with environmental conditions, making it difficult to tease apart the effects of the plant and the environment on biodiversity. Therefore, monitoring programs cannot rely solely on quantifying easy‐to‐measure plant traits but still require estimates of faunal composition when attempting to evaluate ecological state as it pertains to biodiversity.

The high number of rare species and traits observed in the eelgrass meadows studied highlight this habitat's function in hosting transient species, which possibly spend a portion of their life‐cycles in other nearby habitats. Management actions aiming to protect biodiversity should therefore consider protecting whole ecosystems, including networks of diverse habitats, to maximize the benefits for biodiversity and associated services. Such conservation plans may greatly benefit from spatial mapping and monitoring to identify habitats of special interest like eelgrass but also adjacent habitats where species may complete their life‐cycles or escape from predators, ultimately fostering biodiversity more generally. Mapping performed at regular intervals may also help alert drastic changes in meadow size or receding cycles that do not reverse. In this regard, marine protected area (MPA) networks have been recognized as an effective conservation tool for protecting biodiversity in the ocean. For example, the establishment of a network of MPAs in the North Atlantic resulted in a significant increase in regional diversity (γ‐diversity), particularly for species that were previously threatened or endangered (Hays et al., 2020). A network of MPAs can favor connectivity and increase the resilience of marine ecosystems to environmental disturbances (Toonen et al., 2013). The implementation of an MPA network along the coast of France or any other country harboring eelgrass meadows could have long‐reaching benefits for preserving regional diversity and promoting long‐term sustainability.

Eelgrass beds are important marine ecosystems that support a diverse range of associated species. However, the factors that influence the structure and composition of eelgrass‐associated assemblages at different spatial scales are not well understood. This study highlights the importance of considering multiple spatial scales and their associated processes when studying biodiversity associated with eelgrass. The results presented here are in agreement with the prediction that abiotic factors have a greater impact on diversity over larger scales while biotic factors are more prevalent at smaller scales (Pearson & Dawson, 2003). In addition, environmental change can have significant effects on eelgrass‐associated biodiversity, as demonstrated by the cascading effects of the environment on eelgrass and subsequently on assemblage diversity and abundance. Cascading effects can be complex and counteracting through their effects on multiple characteristics of eelgrass. Future work is needed to disentangle the cascading and counteracting effects of the environment on eelgrass and their subsequent effects on biodiversity so that we may better predict change and better protect this diverse marine ecosystem.

## AUTHOR CONTRIBUTIONS

**Alexandre MULLER:** Conceptualization (equal); data curation (lead); formal analysis (lead); methodology (equal); writing – original draft (lead). **Stanislas F. Dubois:** Conceptualization (supporting); formal analysis (supporting); supervision (equal); writing – review and editing (supporting). **Aurelien Boye:** Data curation (supporting); formal analysis (supporting); writing – review and editing (equal). **Ronan Becheler:** Data curation (supporting); methodology (supporting); writing – review and editing (supporting). **Gabin Droual:** Conceptualization (supporting); methodology (lead); writing – review and editing (supporting). **Mathieu Chevalier:** Data curation (supporting); formal analysis (supporting); writing – review and editing (supporting). **Marine Pasquier:** Data curation (supporting); writing – review and editing (supporting). **Loïg Roudaut:** Data curation (supporting); writing – review and editing (supporting). **Jérôme Fournier‐Sowinski:** Conceptualization (supporting); methodology (supporting); writing – review and editing (equal). **Isabelle Auby:** Conceptualization (supporting); methodology (supporting); writing – review and editing (equal). **Flavia L. D. Nunes:** Conceptualization (lead); funding acquisition (supporting); methodology (equal); supervision (lead); writing – original draft (supporting).

## CONFLICT OF INTEREST STATEMENT

The authors declare that the research was conducted in the absence of any commercial or financial relationships that could be construed as a potential conflict of interest.

## Supporting information


Appendix S1

## Data Availability

Data are available on the SEANOE database: https://www.seanoe.org/data/00765/87709/.
