# Supplementary material for: Environmental filtering and biotic interactions act on different facets of the diversity of benthic assemblages associated with eelgrass
Source: Ecol Evol. 2023 Nov 27;13(11):e10159. doi: 10.1002/ece3.10159 (PMC10682608; doi:10.1002/ece3.10159)
Supplement: Supplementary file 1 — Appendix S1 [file ECE3-13-e10159-s001.docx]

**Supplementary Materials**

**Appendix S1: Bibliographic references used for taxonomic identifications of invertebrate fauna associated with eelgrass meadows.**

Gil, J. C. F. (2011). The European fauna of Annelida Polychaeta. 1554. Available at: https://repositorio.ul.pt/handle/10451/4600.

Holdich, D. M., and Jones, J. A. (1983). *Tanaids : keys and notes for the identification of the species*. Linnean Society of London and the Estuarine and Coastal Sciences Association.

Ingle, R. W. (1996). *Shallow-water crabs: keys and notes for identification of the species*. Linnean Society of London and the Estuarine and Coastal Sciences Association.

King, P. E. (1974). *British sea spiders: Arthropoda, Pycnogonida : keys and notes for the identification of the species*. Linnean Society of London and the Estuarine and Coastal Sciences Association.

Lincoln, R. J. (1979). *British marine amphipoda: Gammaridea*. London: British Museum (Natural History).

Naylor, E. E. (1972). *British marine isopods: keys and notes for the identification of the species*. London: Academic Press Inc. Ltd.

San Martín, G. (2003). *Fauna Ibérica. Vol. 21. Annelida polychaeta II: Syllidae*. Consejo Superior de Investigaciones Cientificas.

San Martín, G., and Worsfold, T. M. (2015). Guide and keys for the identification of Syllidae (Annelida, Phyllodocida) from the British Isles (reported and expected species). *Zookeys* 488, 1–29. doi:10.3897/zookeys.488.9061.

Smaldon, G., Hothuis, L. B., and Fransen, C. H. J. M. (1993). *Coastal shrimps and prawns: keys and notes for identification of the species*. Linnean Society of London and the Estuarine and Coastal Sciences Association.

Southward, A. J. (2008). *Barnacles: keys and notes for the identification of British species*. Linnean Society of London and the Estuarine and Coastal Sciences Association.

***Table S1.*** *Biological traits and modalities used in the functional diversity analyses.*

| **Trait** | **Modalities** | **Abbreviation** | **Functions and processes** |
| --- | --- | --- | --- |
| Size | < 10 mm | Very_small | Secondary production, r and k strategies, space utilization (Thrush et al., 2006). |
|  | 10 to 20 mm | Small |  |
|  | 20 to 50 mm | Small_Medium |  |
|  | 50 to 100 mm | Medium |  |
|  | >100 mm | Large |  |
| Feeding mode | Deposit feeder | DF | Resource utilization and availability, nutrient cycling and bentho-pelagic coupling (Thrush et al., 2006). |
|  | Suspension feeder | SF |  |
|  | Grazer | Grazer |  |
|  | Predator/scav | Pred/Scav |  |
| Adult movement | No movement | None | Behavior regarding disturbance, foraging mode, ability to escape predation, migratory requirements, dispersal, fluxes (Queirόs et al., 2013; Solan et al., 2004). |
|  | Within habitat range | Within_habitat |  |
|  | Beyond habitat range | Beyond_home |  |
| Habitat | Tube dweller | Tdw | Sediment reworking potential, species interactions, Biogeochemical requirements, niche creation, refuge, nursery, below sediment oxygenisation. |
|  | Burrow dwelling | Bdw |  |
|  | Attached | Att |  |
|  | Free living | Fl |  |
| Reproduction | Asexual | asx | Colonization potential, r and k strategies, larvae as a source of food, recovery potential, juvenile survival and recruitment success. |
|  | Internal | Int |  |
|  | External  (broadcast spawner) | Ext_Bsp |  |
|  | External (Pseudocopulation) | Ext_Pco |  |
| Development  stage | Direct | Dev_direct | Colonization potential, r and k strategies, larvae as a source of food, recovery and dispersal potential, juvenile survival and recruitment success. |
|  | Indirect - planktotrophic | Dev_plankto |  |
|  | Indirect - lecithotrophic | Dev_lecitho |  |
| Bioturbation | Epifauna | Bioturb_N | Food acquisition, foraging mode, protection against epibenthic and benthopelagic predators, impact on biogeochemistry, organic matter re-distribution, habitat provision demographic control (predation), nutrient cycling (Thrush et al., 2006 , Solan et al., 2004; Queirόs et al., 2013). |
|  | Surficial modifiers | Bioturb_S |  |
|  | Biodiffusors | Bioturb_B |  |
|  | Upward/Downward conveyors | Bioturb_UDC |  |
|  |  |  |  |
| Life span | Short  (< 2 years) | Short_life_span | Large slowing growing species, with longer life spans, to less vulnerable faster growing ones, facing disturbance, longevity increases reproductive successes over time (Cusson and Brouget, 2005). |
|  | Medium  (2 to 5 years) | Medium_life_span |  |
|  | Long  (> 5 years) | Long_life_span |  |

***Table S2:*** *List of explanatory and response variables, with their abbreviations and units, used in this study. Only explanatory variables kept after the removal of collinear variables, identified using Spearman correlation coefficient, are presented here. Stepwise selection procedure was performed within each response and explanatory variables (*Zostera marina *traits and Environmental variable). Variables included within the pSEM are shown in bold.*

|  | Abbreviation | Description | Units |
| --- | --- | --- | --- |
| **Response variables** | |  |  |
| *Zostera marina* traits | Leaf_weigth | Leaf biomass per square meter | g.m-2 |
|  | Sheath_weight | Sheath biomass per square meter | g.m-2 |
|  | Rhizome.Root_weight | Underground biomass per square meter | g.m-2 |
|  | Leaf.Sheath.Rhizome.Root.ratio | Ratio above to underground biomass | / |
|  | Density.m2 | Mean shoot density per square meter | shoot.m-2 |
|  | Sheath_height | Mean sheath height | mm |
|  | **Leaf length** | **Mean leaf length** | **mm** |
|  | **Leaf width** | **Mean leaf width** | **mm** |
|  | Leaf area index (LAI) | Average leaf surface area per shoot (leaf length times leaf width), multiplied by the shoot density |  |
|  | No.leaf_per_shoot | Number of leaves per shoot | / |
|  | Broken_apex | Percent of broken leaves | % |
| **Explanatory variable** | |  |  |
| Substrate | Sand | Sediment fraction between 63µm and  1mm | % |
|  | mud | Sediment fraction < 63µm | **%** |
|  | Trask(So) | Sorting index | / |
|  | D50 | Mean grain size | µm |
|  | OM | Organic matter content | % |
| Hydrology | Suspensed_organic_particule_matter | Mean suspended organic particulate matter | / |
|  | **Temperature** | **Mean sea water temperature** | **°C** |
|  | SAL | Mean salinity | **‰** |
| Exposure | **Current_velocity** | **Mean current velocity** | **m.s-1** |
| Tidal | **Tidal_amplitude** | **Mean delta water level** | **m** |

**Table S3** Path coefficients and p-values for the structural equation model presented in Figure 6. For variable abbreviations, see Table S2. By default, each line of the table represents a directed relationship between two variables (arrows in Figure 6). Lines in the tables where the variables start by “~~” represent correlated errors (double-headed arrows in Figure 6). Correlated errors represent unmeasured sources of variance that are influencing the relationship between two variables, without imparting causality.

| **Response** | **Predictor** | **Estimate** | **Std.**  **Error** | **Crit.**  **Value** | **p-value** | **Std.**  **Estimate** |
| --- | --- | --- | --- | --- | --- | --- |
| Rhizome.Root_weight | Temperature | 0.22 | 0.05 | 4.03 | <0.01 | 0.47 |
| Leaf_length | Temperature | 96.73 | 19.57 | 4.94 | <0.01 | 0.54 |
| Leaf_width | Temperature | 1.40 | 0.29 | 4.79 | <0.01 | 0.54 |
| Leaf_width | Tidal_amplitude | -0.43 | 0.14 | -3.07 | <0.01 | -0.49 |
| Leaf_width | Current_velocity | 16.57 | 8.15 | 2.03 | 0.05 | 0.27 |
| Abundance | Rhizome.Root_weight | -2378.14 | 1018.94 | -2.33 | 0.02 | -0.3 |
| Abundance | Leaf_length | -6.94 | 3.5 | -1.99 | 0.05 | -0.34 |
| Abundance | Leaf_width | 1359.87 | 282.19 | 4.82 | <0.01 | 0.95 |
| Abundance | Tidal_amplitude | 614.89 | 186.64 | 3.29 | <0.01 | 0.5 |
| Richness | Leaf_length | -0.02 | 0.00 | -3.97 | <0.01 | -0.53 |
| Richness | Leaf_width | 1.48 | 0.48 | 3.11 | <0.01 | 0.62 |
| Richness | TEMP | 2.27 | 0.94 | 2.40 | 0.02 | 0.37 |
| Richness | Tidal_amplitude | 2.08 | 0.25 | 8.32 | <0.01 | 1.00 |
| FRic | Rhizome.Root_weight | -0.07 | 0.06 | -1.19 | 0.24 | -0.13 |
| FRic | Leaf_length | 0.00 | 0.00 | -3.07 | <0.01 | -0.42 |
| FRic | Leaf_width | 0.08 | 0.02 | 4.14 | <0.01 | 0.82 |
| FRic | Temperature | 0.08 | 0.04 | 2.05 | 0.05 | 0.32 |
| FRic | Tidal_amplitude | 0.09 | 0.01 | 7.94 | <0.01 | 0.97 |
| ~~Richness | ~~FRic | 0.52 | - | 4.65 | <0.01 | 0.52 |
| ~~Rhizome.Root_weight | ~~Leaf_length | 0.28 | - | 2.17 | 0.02 | 0.28 |
| ~~Leaf_width | ~~Leaf_length | 0.62 | - | 6.01 | <0.01 | 0.62 |
| ~~Rhizome.Root_weight_per_shot | ~~Leaf_width | 0.16 | - | 1.2 | 0.12 | 0.16 |
| ~~Richness | ~~PC1 | -0.39 | - | -3.18 | <0.01 | -0.39 |
| PC1_abundance | Rhizome.Root_weight | -0.06 | 0.02 | -2.44 | 0.02 | -0.14 |
| PC1_abundance | Temperature | -0.06 | 0.02 | -3.84 | <0.01 | -0.32 |
| PC1_abundance | Current_velocity | 4.60 | 0.43 | 10.73 | <0.01 | 1.00 |
| PC1_abundance | Tidal_amplitude | -0.11 | 0.01 | -15.05 | <0.01 | -1.66 |
| PC2_abundance | Leaf_length | 0 | 0 | -2.08 | 0.04 | -0.14 |
| PC2_abundance | Rhizome.Root_weight | -0.07 | 0.02 | -3.53 | <0.01 | -0.18 |
| PC2_abundance | Leaf_width | 0.03 | 0.01 | 3.51 | <0.01 | 0.38 |
| PC2_abundance | Abundance | 0 | 0 | 1.49 | 0.14 | 0.07 |
| PC2_abundance | Temperature | -0.24 | 0.01 | -17.34 | <0.01 | -1.35 |
| PC2_abundance | Tidal_amplitude | -0.05 | 0.01 | -6.88 | <0.01 | -0.79 |
| PC2_abundance | Current_velocity | 1.13 | 0.34 | 3.31 | <0.01 | 0.27 |

*
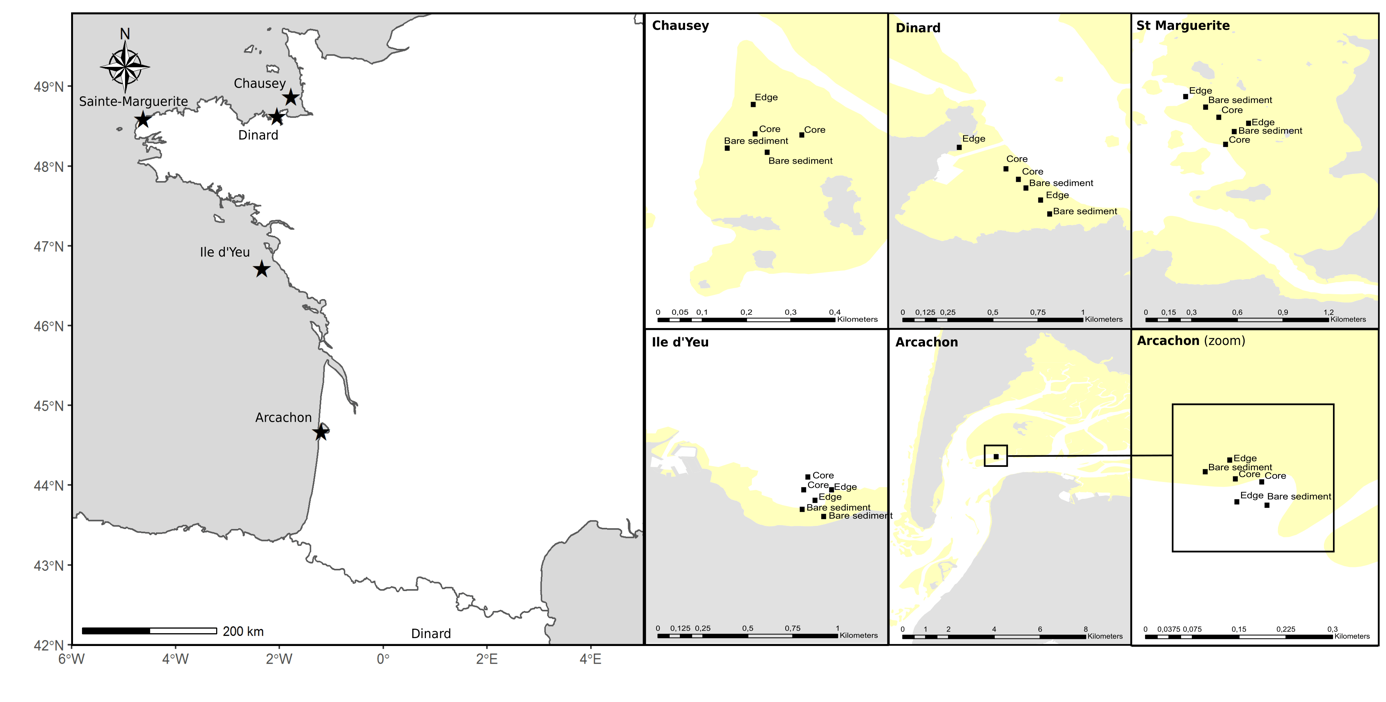
***Figure S1.** Map indicating the locations of the 5 study sites of *Zostera marina* meadows in France: three in the English Channel and two in the Bay of Biscay. All sites were sampled at two stations for three different habitat types: core, edge and bare sediment, for a total of six sampling stations per site. Yellow areas correspond to the intertidal zone.


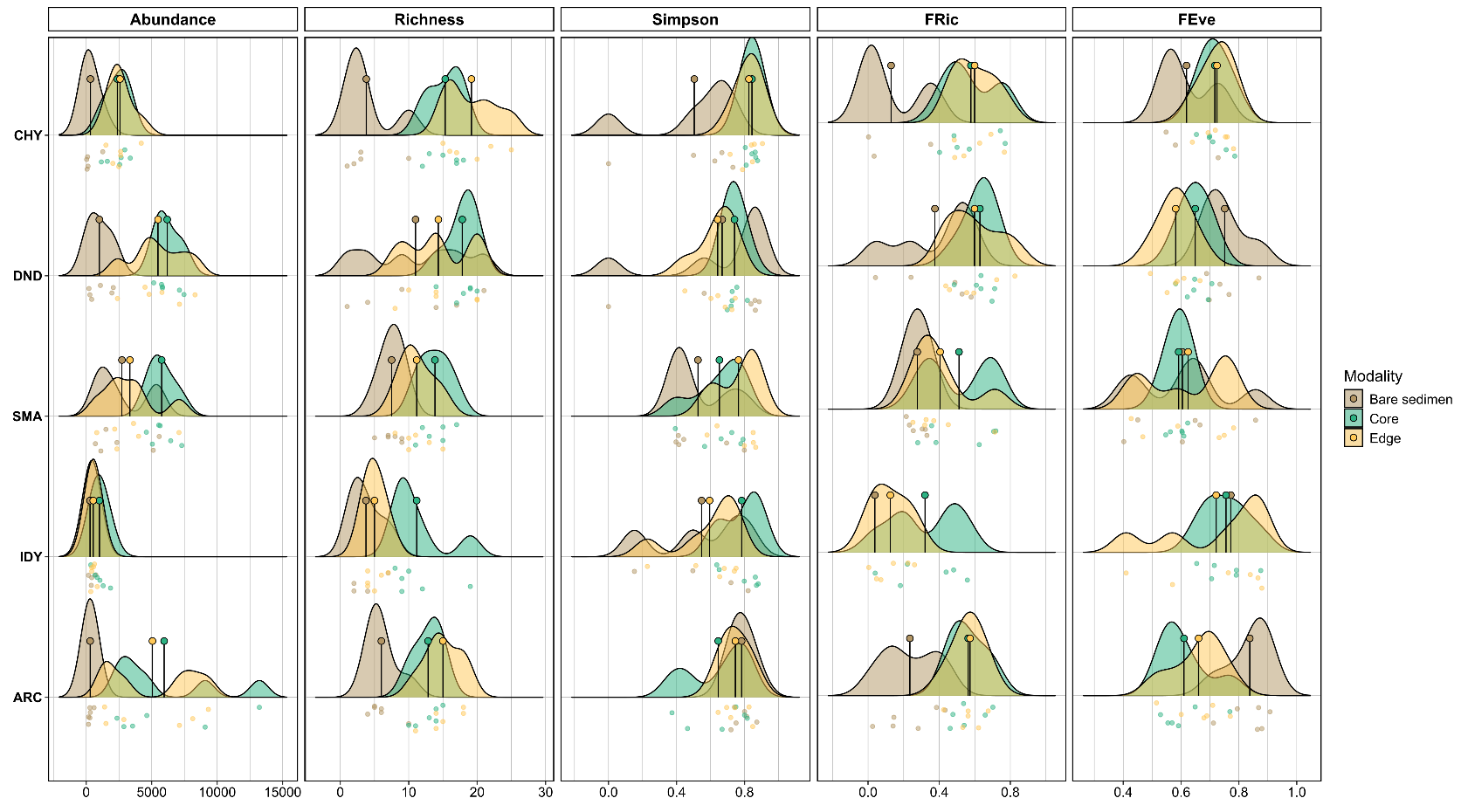
**Figure S2.** Distribution of taxonomic and functional α‐diversity indices among the five sites: Chausey (CHY), Dinard (DND), Sainte-Marguerite (SMA), Ile d’Yeu (IDY), Arcachon (ARC). For each site, the distributions include the values of the different meadow parts sampled. The mean value for each of these indices is represented by the point pinned on each distribution. Abundance corresponds to the total abundance of each assemblage. Richness corresponds to the taxonomic richness of the assemblage. Simpson corresponds to Simpson's diversity index. FRic, FEve correspond to the functional richness and the functional evenness, respectively, and were calculated on 3 PCOA axes representing 42% of the original species dissimilarity matrix.

**Figure S3.** Venn diagram indicating the number of species shared between and among the five sampled meadows and unique to each meadow.

***
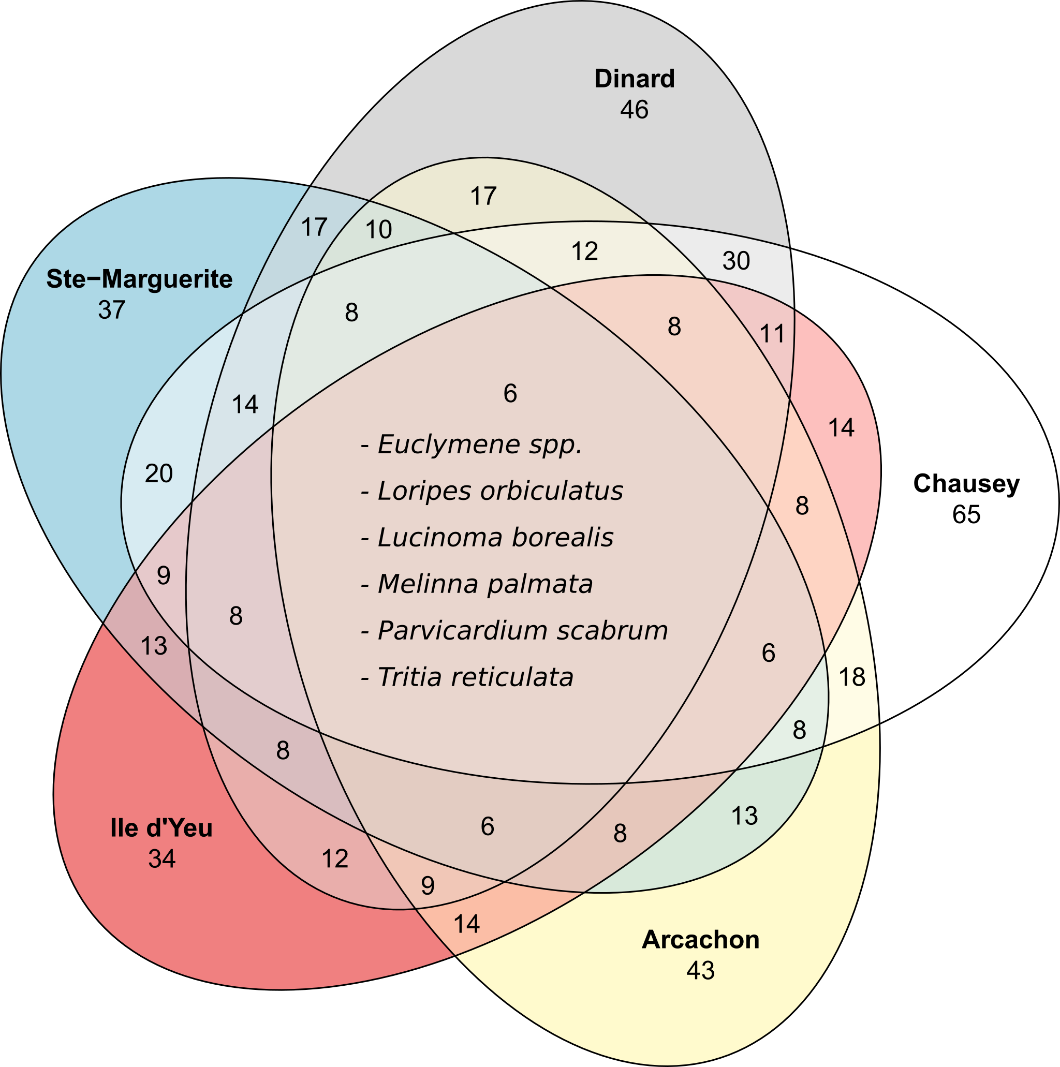
***


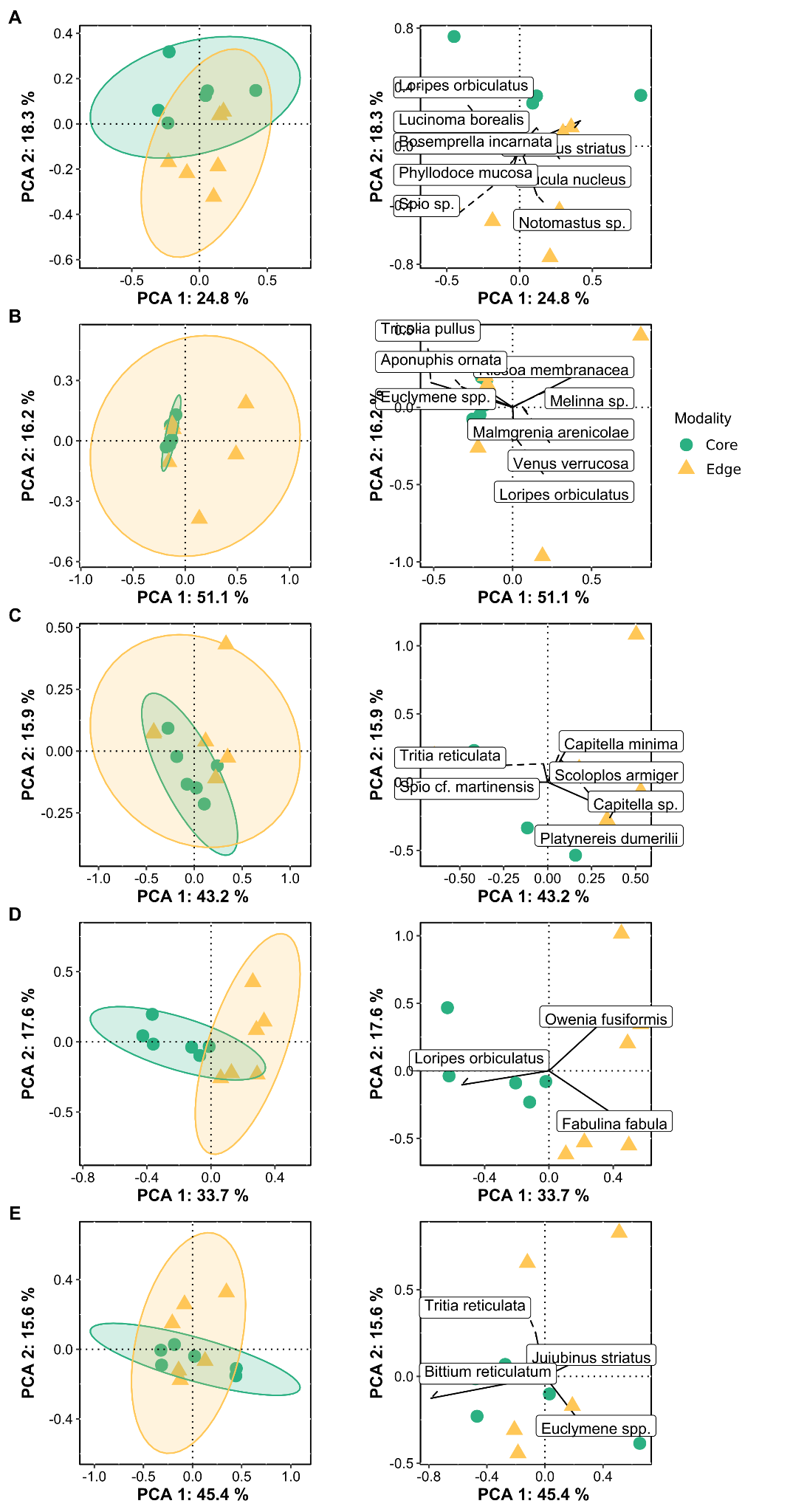
**Figure S4.** Principal component analysis of the Hellinger-transformed abundance of taxa among the five *Zostera marina* beds in core and edge habitats. **A,** Chausey **B,** Dinard **C,** Saite-Marguerite **D,** Ile d’Yeu **E,** Arcachon. The sites for each point sampled core and edge with their 95% confidence dispersion ellipses, represented in scaling 1 (distance biplot) preserving the distances among the sites. Within-site dispersions represent variation of the communities among the two habitats.

***
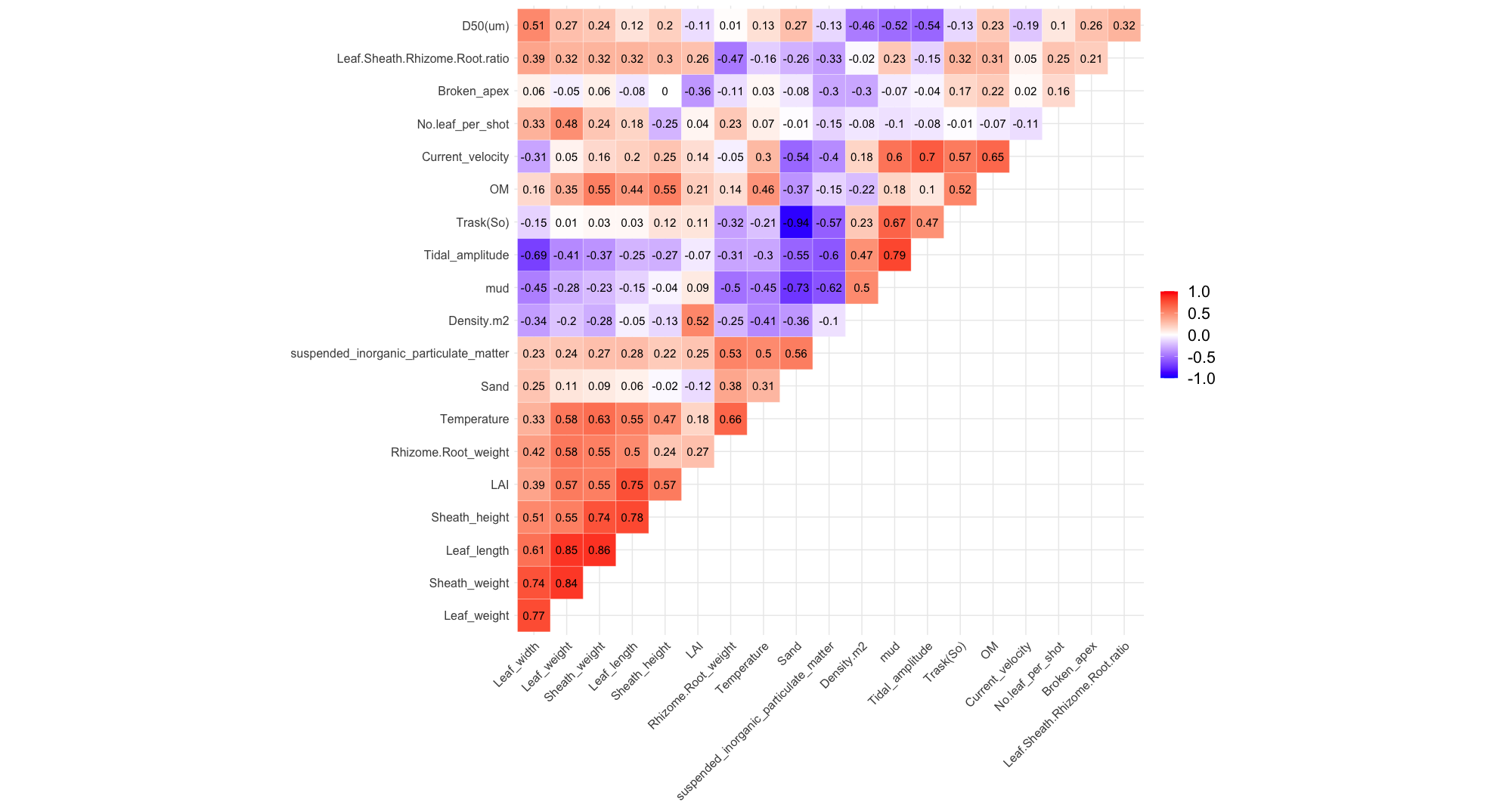
*Figure S5.** Spearman cross-correlation matrix of the metrics in Table S2 with significance levels (blue for negative and red for positive correlation). Correlations with p-values > 0.05 are considered non-significant. In this case the correlation coefficient values are left blank.

*
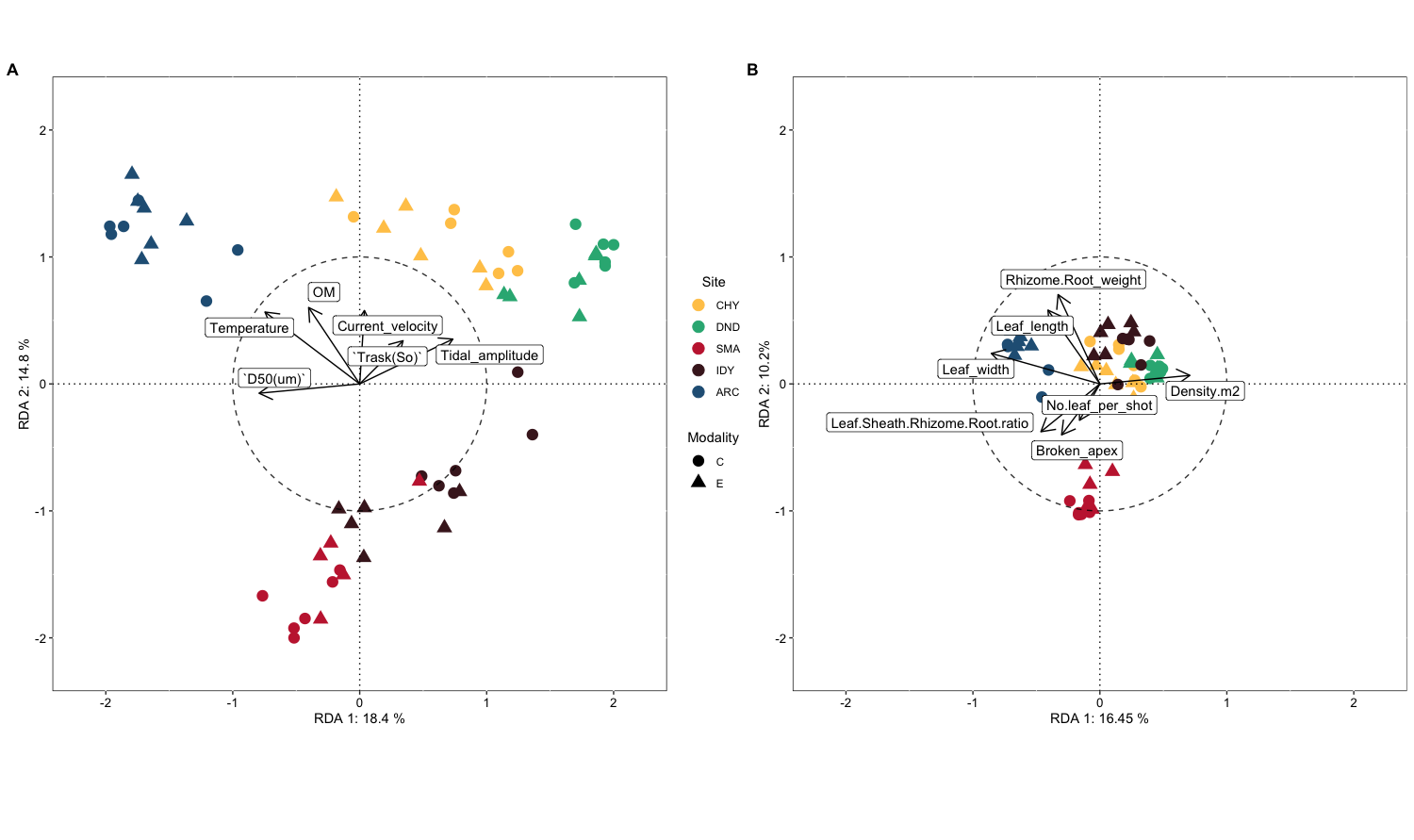
***Figure S6.** Redundancy analysis of the Hellinger-transformed abundance of taxa associated with the five *Zostera marina* beds sampled on two habitat types (core and edge) against **A.** the environmental variables selected using stepwise selection based on adjusted R² (scaling type 2); and **B.** the *Zostera marina* traits selected using stepwise selection based on adjusted R² (scaling type 2).
